# Their Place in the War Industries To-Day

**Published:** 1917-12-15

**Authors:** 


### HOSPITALS AND THE CHRISTMAS TRADITION.

### Their Place in the War Industries To-day.

A year ago the position of the Allies gave less hope of approaching victory than is happily the case to-day. America was still neutral, the enemy's grip on Northern France had not begun to weaken, the Eastern fronts showed no advance in Palestine to set off the memory of our reverses, and the phase of trench warfare in Flanders seemed interminable. Yet a far-seeing preacher, who had not lost his sense of proportion, took as his text last Christmas morning the words "Rejoice and be exceeding glad," and explained to a depressed, but attentive, audience that Christmas was the symbol of a great religious truth which was as much beyond the power of circumstance as the return of spring. It was a fine sermon on a very simple theme, and is still treasured by some who heard it. In the buzz and floundering of the lay newspapers, in the conflicting panic of manufactured public opinion, it asserted a principle in the presence of which this chaos assumes its proper proportion as a symptom of ignorance and nerves. The work of every day and every week is not controlled by such manifestations, and the faith which inspires such work is not rooted in the sand of such confusion.

### The Hospitals' Special Claim.

Therefore the voluntary hospitals, which represent the powers of men fighting against destruction, still pursue the victories of peace in the middle of war. For war is the abuse of scientific knowledge. Even the cannon has its function in peace—namely, that of locomotion. For the internal combustion engine is only a gun which drives the wheels. But hospital work cannot be so abused. Therefore hospitals assert, without equivocation or apology, their claim to special support this Christmas.

They do so, in fact, with particular assurance, because high prices and high wages do not neutralise each other. Put simply, the fact is that the more money which passes through any one's pocket the better off such a person will be. His income is higher, his expenses are greater, but in each case he is dealing with larger sums of money. Therefore he is able, not only to spend more, but to save more. This, which was one of the attractions of life in the Colonies before the war, is now one of the advantages of living in England. The roots of this war prosperity are indeed artificial, but the prosperity for the present is a fact. Therefore the voluntary hospitals appeal with great cheerfulness for an extra share of the public's generosity, and the old excuse that the public cannot afford to give is simply laughed out of countenance.

Since it is the business of hospitals to spend, and not to save anything except the lives or limbs of their patients, the war prosperity only touches them in so far as it prompts an easy flow of coin from their supporters. On the rise in prices, which they feel, it is unnecessary to dwell. The work which they are doing for civilians and soldiers is also common knowledge. But their work for civilians and soldiers is not all.

In the Midlands and the North there is a huge industrial army of workers in Government factories engaged upon the production of munitions and aeroplanes. This broad division of the work does not exhaust it. Modern industry depends upon specialisation, and the complete gun or the finished aeroplane is a combination of many parts, on each of which is engaged an army of workers. It is the same with the shipping industry. It is the same even with army clothing. Each of the divisions of workers, which is recruited by men and women, is liable to special risks of its own. Each of these risks is attended by its incidental accidents. As the industrial army grows, the demand upon the hospitals grows with it, and it is now a recognised fact that those in the factories at home are by no means free from risk or injury. The voluntary hospitals are therefore an essential part of the vast machinery of war, on which the soldiers and their families and the industrial army and its families alike rely for treatment and restoration.

### A Thank-Offering From the Healthy.

To meet the cost of this patriotic work the voluntary hospitals have been busy organising subscriptions from the workers on similar lines to those which have grown familiar through the success of workmen's subscriptions in the ordinary industries of peace. But these subscriptions have never borne, nor been intended to bear, the whole cost of the hospital treatment. The voluntary system, which seems chaotic to foreigners, has rested on the principle that those who are well owe a duty of personal service to their sick fellows, among whom at any moment some mischance may thrust them. Therefore the appeal of the hospitals has not been to any class of patient but simply to those who are well. They ask for a thankoffering from the healthy; they are not businesses which have something to sell, a premium as it were, in advance. There is no doubt that this public-spirited atmosphere has made hospitals happy places, and has set the patients' interests above the interests of science or of professors, and has brought to the great number of voluntary workers on whom each hospital relies the reward which belongs to those who have an important public cause to forward. It is therefore on something larger than sentiment, and stronger than pathos, that the hospitals' claim rests. It rests upon a great work, and a fine tradition, for it is supported not by the chance impulse of casual generosity, but by the living gospel of personal service, which the war has now taught to the whole nation. It was first preached by the hospitals in peace-time, and is the central lesson of Christmas, which celebrates the birth of One who came to take our nature upon Him, and to teach that greatness means personal service and nothing else.

